# The SARS-CoV-2 RNA polymerase is a viral RNA capping enzyme

**DOI:** 10.1093/nar/gkab1160

**Published:** 2021-11-29

**Authors:** Alexander P Walker, Haitian Fan, Jeremy R Keown, Michael L Knight, Jonathan M Grimes, Ervin Fodor

**Affiliations:** Sir William Dunn School of Pathology, University of Oxford, South Parks Road, Oxford OX1 3RE, UK; Sir William Dunn School of Pathology, University of Oxford, South Parks Road, Oxford OX1 3RE, UK; Division of Structural Biology, Wellcome Centre for Human Genetics, University of Oxford, Oxford OX3 7BN, UK; Sir William Dunn School of Pathology, University of Oxford, South Parks Road, Oxford OX1 3RE, UK; Division of Structural Biology, Wellcome Centre for Human Genetics, University of Oxford, Oxford OX3 7BN, UK; Diamond Light Source Ltd, Harwell Science & Innovation Campus, Didcot OX11 0DE, UK; Sir William Dunn School of Pathology, University of Oxford, South Parks Road, Oxford OX1 3RE, UK

## Abstract

SARS-CoV-2 is a positive-sense RNA virus responsible for the Coronavirus Disease 2019 (COVID-19) pandemic, which continues to cause significant morbidity, mortality and economic strain. SARS-CoV-2 can cause severe respiratory disease and death in humans, highlighting the need for effective antiviral therapies. The RNA synthesis machinery of SARS-CoV-2 is an ideal drug target and consists of non-structural protein 12 (nsp12), which is directly responsible for RNA synthesis, and numerous co-factors involved in RNA proofreading and 5′ capping of viral RNAs. The formation of the 5′ 7-methylguanosine (m^7^G) cap structure is known to require a guanylyltransferase (GTase) as well as a 5′ triphosphatase and methyltransferases; however, the mechanism of SARS-CoV-2 RNA capping remains poorly understood. Here we find that SARS-CoV-2 nsp12 is involved in viral RNA capping as a GTase, carrying out the addition of a GTP nucleotide to the 5′ end of viral RNA via a 5′ to 5′ triphosphate linkage. We further show that the nsp12 NiRAN (nidovirus RdRp-associated nucleotidyltransferase) domain performs this reaction, and can be inhibited by remdesivir triphosphate, the active form of the antiviral drug remdesivir. These findings improve understanding of coronavirus RNA synthesis and highlight a new target for novel or repurposed antiviral drugs against SARS-CoV-2.

## INTRODUCTION

Coronaviruses pose a serious threat to human health as they can cause severe respiratory disease and have pandemic potential. Severe acute respiratory syndrome coronavirus (SARS-CoV) was responsible for an epidemic in 2003 which caused nearly 800 deaths, and SARS-CoV-2 is responsible for the ongoing Coronavirus Disease 2019 (COVID-19) pandemic (1). Therefore, understanding the coronavirus life cycle in order to develop novel therapeutics is of utmost importance.

SARS-CoV-2 is a betacoronavirus in the order *Nidovirales*, and it has a positive-sense RNA genome of around 30 kb (1,2). Two-thirds of the viral genome encode two overlapping open reading frames (ORFs), 1a and 1b, which are translated immediately upon infection. The resulting polyproteins are cleaved to produce non-structural proteins (nsps) 1–16, which collectively form the membrane-associated replication-transcription complex (RTC). The RTC is responsible for synthesising new full-length viral genomes as well as subgenomic RNAs, which contain the ORFs of viral structural and accessory proteins (3–5).

Coronavirus genomes are thought to have a 5′ 7-methylguanosine (m^7^G) cap, based on indirect evidence such as the presence of virally-encoded capping enzymes and some direct biochemical evidence (4,6–8). Synthesising m^7^G capped RNA requires several distinct catalytic activities, most of which have already been identified in the RTC. Specifically, nsp13, nsp14 and nsp16 are involved in m^7^G cap synthesis as a 5′ triphosphatase, N7-methyltransferase, and 2’-*O*-methyltransferase, respectively (9–12). m^7^G cap synthesis pathways also require a guanylyltransferase (GTase) enzyme to covalently link GTP to the 5′ end of an RNA substrate. This activity has recently been demonstrated for nsp12, the RNA-dependent RNA polymerase (RdRp) component of the RTC, using GTP or UTP nucleotides (13).

The C-terminal RdRp domain of SARS-CoV-2 nsp12 is structurally similar to other viral RdRp proteins, making it a key target for repurposed nucleotide analogue drugs (14–16). At the N-terminus of nsp12 is a 250-amino acid residue nidovirus RdRp-associated nucleotidyltransferase (NiRAN) domain, which has a number of proposed functions based on its ability to covalently bind to nucleotides (17–20). Nsp12 is thought to be a core component of the RTC, and must interact with its accessory proteins nsp7 and nsp8 to function as a processive RNA polymerase (21). Nsp12 also interacts directly with nsp13 and nsp14 which, in addition to their roles in m^7^G cap synthesis, function as an RNA helicase and a proofreading exonuclease, respectively (22–25). These interactions may therefore be important to facilitate viral RNA synthesis or co-ordinate capping.

Despite its importance in the viral life cycle, the mechanism of coronavirus RNA capping remains poorly understood. Here we provide insight into this process by demonstrating that the NiRAN domain of SARS-CoV-2 nsp12 is a GTase enzyme involved in viral m^7^G cap synthesis. We also show that this activity can be inhibited by remdesivir triphosphate, highlighting the NiRAN domain as a possible target for repurposed antiviral drugs.

## MATERIALS AND METHODS

### SARS-CoV-2 growth and reverse transcription-qPCR

SARS-CoV-2 England/02/2020 isolate was received from Public Health England Virus Reference Laboratory at P1 and propagated in Vero CCL-81 cells (ATCC) in containment level (CL) 3 to produce P3 virus stock, confirmed identical by sequencing. 8.1 × 10^5^ Vero CCL-81 cells in Dulbecco's modified Eagle's medium, 1% fetal calf serum, 1× Pen-Strep and 1× GlutaMAX (Thermo) were mixed with SARS-CoV-2 England/02/2020 (P3) at MOI 1.0 and seeded in each 35mm well of a 6- well culture dish with 2 ml culture medium. At 3, 6, 9 and 12 h post-infection medium was removed and 1 ml TRI reagent (Sigma) was added to the wells, then total cellular RNA extraction and precipitation was performed according to the manufacturer's instructions. Mock infected samples were also included as a negative control.

70 ng of extracted RNA (quantified on a NanoDrop 2000 Spectrophotometer) was annealed to 1 μM each reverse primer in 5 μl mixtures, by heating to 95°C and cooling to room temperature. Reverse primer sequences (purchased from Sigma) are as follows: Full-length, 5′-AATTAGTTATTAATTATACTGCGTG-3′; S, 5′-GCAGGGGGTAATTGAGTT-3′; 3a, 5′-GCGCGAACAAAATCTGAA-3′; E, 5′-CGCTATTAACTATTAACGTACCT-3′; M, 5′-GCTCTTCAACGGTAATAGTAC-3′; 6, 5′-CCAATCCTGTAGCGAC-3′; 7a, 5′-AAAAGTACTGTTGTACCTCT-3′; 8, 5′-TGAGTACATGACTGTAAACTAC-3′; N, 5′-GTTCTCCATTCTGGTTACTG-3′; GAPDH mRNA, 5′-CAAAGTTGTCATGGATGACC-3′; 5S rRNA, 5′-TCCCAGGCGGTCTCCCATCC-3′. Reverse transcription was carried out by addition of 50 U SuperScript III (Invitrogen), 1× first-strand buffer (Invitrogen), 10 U RNasin (Promega) and 10 mM dithiothreitol (DTT) to a total reaction volume of 10 μl. Reactions were incubated at 37°C for 15 min, 50°C for 45 min and then 70°C for 10 min. cDNA was stored at −20°C prior to qPCR analysis. To perform qPCR 1 μl of the resulting cDNA was mixed with 1× qRT-PCR Brilliant III SYBR Master Mix (Agilent) and a further 0.75 μM of the reverse primer, as well as 0.75 μM of the corresponding forward primer in a total reaction volume of 10 μl. Forward primer sequences (purchased from Sigma) are as follows: Leader (used for all viral RNAs), 5′-ATTAAAGGTTTATACCTTCCCAG-3′; GAPDH mRNA, 5′-CCATGGAGAAGGCTGGGG-3′; 5S rRNA, 5′-GTCTACGGCCATACCACCCTGAACG-3′. qPCR was performed in 384-well skirted reaction plates (Alpha Laboratories) on a Quantstudio 5 RT-PCR machine (Applied Biosystems) with the following cycling conditions: 95°C 10 min; 95°C 15 s, 60°C 30 s, 60 cycles; melt curve analysis. qPCR data analysis was performed using the included Design and Analysis Software v1.5.2 (Applied Biosystems). Two technical replicates of qPCR were performed per sample and averaged. Data presented were normalised to the GAPDH mRNA Ct value

### RNA immunoprecipitation

2 μg total cellular RNA extracted from Vero cells 9 h post-infection with SARS-CoV-2 (see above) was mixed with 2 μg mouse anti-m^7^G antibody (MBL; cat. RN016M) or mouse anti-his antibody (R&D Systems; cat. MAB050) in a total volume of 200 μl wash buffer (1× phosphate-buffered saline (PBS), 0.01% bovine serum albumin, 2 mM EDTA, 1mM DTT and 0.1 U/μl RNasin). 50 μl washed Sheep Anti-Mouse IgG Dynabeads (Invitrogen) were added and incubated with the mixture at room temperature for 30 min. Beads were then washed 3x in 1 ml wash buffer and RNA was eluted by addition of 1 ml TRI reagent. RNA extraction was then performed according to the manufacturer's instructions, except that 20 μg glycogen (Roche) was added to aid RNA precipitation. Input and eluted RNA was analysed using RT-qPCR as described above. Data presented were normalized to the Ct value of 5S rRNA, and then the input sample was set to 0. Data were analysed using Prism 9 (GraphPad).

### SARS-CoV-2 protein expression and purification

The full-length nsp12 gene from SARS-CoV-2 was cloned into the MultiBac system with a Tobacco Etch Virus (TEV) protease cleavable protein-A tag at the C-terminus (26). Nsp12 was expressed in Sf9 insect cells and initial purification was performed by affinity chromatography as previously described for the influenza virus RNA polymerase, but with minor modifications: all buffers were supplemented with 0.1 mM MgCl_2_ and the NaCl concentration was changed to 300 mM (27). After overnight cleavage with TEV protease, released protein was further purified on a Superdex 200 Increase 10/300 GL column (GE Healthcare) using 25 mM HEPES–NaOH, pH 7.5, 300 mM NaCl and 0.1 mM MgCl_2_. Fractions containing nsp12 were pooled, concentrated, and stored at 4°C.

The NiRAN construct (nsp12 amino acid residues 1–259) and full-length nsp7 and nsp8 genes from SARS-CoV-2 were cloned into pGEX-6P-1 vector (GE Healthcare) with an N-terminal GST tag followed by a PreScission protease site. Proteins were expressed in *Escherichia coli* BL21 (DE3) cells, then purified on Glutathione Sepharose resin (GE Healthcare). After overnight cleavage with PreScission protease, the released proteins were further purified on a Superdex 75 Increase 10/300 GL column (GE Healthcare) using 25 mM HEPES–NaOH, pH 7.5, 300 mM NaCl and 0.1 mM MgCl_2_. Fractions containing target proteins were pooled, concentrated, and stored at 4°C.

The full-length nsp14 gene from SARS-CoV-2 was cloned into a pGEX-6P-1 vector (GE Healthcare) with an N-terminal GST tag followed by a PreScission protease site. The plasmid was transformed into *E. coli* BL21 (DE3) cells, then 5 ml of overnight culture was used to inoculate 1 l of terrific broth (TB) supplemented with 100 mg/l ampicillin and 10 μM ZnCl_2_. The culture was grown to an OD_600_ of 2, then induced with 1mM IPTG and incubated at 18°C for 16 h. Cells were harvested and resuspended in a buffer containing 50 mM Tris pH 8, 500 mM NaCl, 5% (v/v) glycerol, 1 mM TCEP, 1× protease inhibitor (Roche) and 10 mg of lysozyme, then sonicated. Lysate was clarified by centrifugation, applied to Glutathione Sepharose resin and incubated for 3 h before washing 3× in a buffer containing 25 mM HEPES pH 7.5, 500 mM NaCl, 5% (v/v) glycerol and 1 mM DTT. After overnight cleavage with PreScission protease, eluate was concentrated before further purification on a Superdex 200 Increase 10/300 GL column using 20 mM HEPES pH 7.5, 150 mM NaCl, 1 mM DTT. Fractions containing nsp14 were pooled, concentrated, and stored at −80°C

### 5′ Triphosphatase activity assays

Purified SARS-CoV-2 nsp13 with an N-terminal His6-ZBasic tag in 25 mM HEPES–NaOH, pH 7.5, 300 mM NaCl and 5% glycerol buffer was a kind gift from Yuliana Yosaatmadja and Opher Gileadi. 250 nM nsp13 was incubated with 5 mM MgCl_2_, 4.75 μM ATP and 0.25 μM γ-^32^P-ATP for the indicated time at 30°C, then reactions were stopped by addition of 80% formamide and 10 mM EDTA, followed by heating to 95°C for 3 min. Inactivated nsp13 was heated to 70°C for 5 min prior to incubation. As a positive control, 0.5 U/μl FastAP Thermosensitive Alkaline Phosphatase (Thermo) was incubated with 4.75 μM ATP and 0.25 μM γ-^32^P-ATP for 1 h at 37°C. Reaction products were resolved by 20% denaturing PAGE with 7 M urea, and visualized by phosphorimaging on a Fuji FLA-5000 scanner. Data were analysed using ImageJ and Prism 9 (GraphPad).

To generate diphosphorylated RNA for GTase reactions 5 μM 20mer model RNA (5′-AAUCUAUAAUAGCAUUAUCC-3′), purchased as a 1:1 mixture of di- and triphosphorylated RNA (Chemgenes), was incubated with 250 nM nsp13 in the presence of 5 mM MgCl_2_ for 5 min at 30°C, followed by heat inactivation at 70°C for 5 min. The resulting diphosphorylated RNA stock was used for all GTase reactions.

### Guanylyltransferase activity assays

Where indicated, 5 μM diphosphorylated 20mer RNA substrate (see above) was pre-treated with 0.3 U/μl FastAP Thermosensitive Alkaline Phosphatase (Thermo) at 37°C for 1 h. Alternatively, 5 μM diphosphorylated 20mer RNA substrate was pre-treated with 0.5 U/μl RNA 5′ Pyrophosphohydrolase (NEB) in 1× NEBuffer 2 (NEB) at 37°C for 1 h. Enzymes were heat inactivated at 75°C for 5 min before the resulting RNA substrates were used in GTase reactions.

To run the GTase reaction, 500 nM SARS-CoV-2 nsp12 or NiRAN domain was incubated with 1 μM diphosphorylated or phosphatase-treated 20mer RNA, 0.05 μM α-^32^P-UTP or α-^32^P-GTP, 5 mM MgCl_2_, 10 mM KCl, 1 U/μl RNasin and 1 mM DTT in a 3 μl reaction at 30°C for 240 min unless stated otherwise. Where indicated, nsp7 and/or nsp8 were included at a 5:1 excess over nsp12. GTase reactions involving vaccinia capping enzyme were run for 60 min under the same conditions, using 0.01 U/μl vaccinia capping enzyme (NEB) instead of SARS-CoV-2 protein.

Where indicated, completed GTase reactions were treated with 0.3 U/μl FastAP Thermosensitive Alkaline Phosphatase (Thermo) at 37°C for 1 h. Alternatively, reactions were treated with 0.5 U/μl RNA 5′ Pyrophosphohydrolase (NEB) in 1× NEBuffer 2 (NEB) at 37°C for 1 h. All reactions were stopped by addition of 80% formamide and 10 mM EDTA, followed by heating to 95°C for 3 min. Reaction products were resolved by 20% denaturing PAGE with 7 M urea, and visualized by phosphorimaging on a Fuji FLA-5000 or Typhoon FLA-9500 scanner. Data were analysed using ImageJ and Prism 9 (GraphPad).

### RNA polymerase activity assays

Nsp7, nsp8 and nsp12 were mixed at a molar ratio of 5:5:1 to form the nsp7/8/12 complex. Activity assays were performed essentially as described for SARS-CoV nsp7/8/12 (21). Briefly, 40mer LS1 (5′-CUAUCCCCAUGUGAUUUUAAUAGCUUCUUAGGAGAAUGAC-3′) and 5′ ^32^P-radiolabelled 20mer LS2 (5′-GUCAUUCUCCUAAGAAGCUA-3′) RNAs corresponding to the 3′ end of the SARS-CoV genome (without the polyA tail) were pre-annealed by heating to 70°C for 5 mins followed by cooling to room temperature. 50 nM pre-annealed RNA was incubated for the indicated time at 30°C with 500 nM nsp7/8/12 complex, in a 3 μl reaction containing 5 mM MgCl_2_, 0.5 mM of each ATP, UTP, GTP and CTP, 10 mM KCl, 1 U/μl RNasin and 1 mM DTT. Remdesivir triphosphate (MedChemExpress) was included where indicated, and for these experiments the ATP, UTP, GTP and CTP concentration was reduced to 1 μM. Reactions were stopped by addition of 80% formamide and 10 mM EDTA, followed by heating to 95°C for 3 min. Reaction products were resolved by 20% denaturing PAGE with 7 M urea, and visualized by phosphorimaging on a Fuji FLA-5000 scanner. Data were analysed using ImageJ and Prism 9 (GraphPad).

### 7-Methylguanosine synthesis assays

SARS-CoV-2 nsp12 GTase activity assays were set up as described above, except that 1 mM GTP was used instead of α-^32^P-GTP, the diphosphorylated RNA concentration was increased to 10 μM and the reaction volume was increased to 5 μl. Reactions were incubated at 30°C for 240 min then heated to 95°C for 3 min. 500 nM SARS-CoV-2 nsp14 and 0.1 mM *S*-adenosylmethionine (NEB) were then added and reactions were incubated at 30°C for a further 60 min, then heated to 95°C for 3 min.

Reaction mixtures were dotted onto nylon membrane and allowed to air dry for 10 min, then heated to 80°C for 10 min under a light vacuum. Membranes were blocked in 1× PBS, 0.1% Tween-20 (Sigma) and 2.5% skimmed milk powder (Marvel), then incubated with mouse anti-m^7^G primary antibody (MBL; cat. RN016M) followed by goat anti-mouse HRP-conjugated secondary antibody (Sigma; cat. A4416) in blocking buffer. Membranes were washed 3× in 1× PBS, 0.1% Tween-20 then HRP activity was detected using Immobilon Western Chemiluminescent HRP Substrate (Millipore) and exposure on Super RC Fuji Medical X-ray film (Kodak). Data were analysed using ImageJ and Prism 9 (GraphPad).

## RESULTS

### Genomic and subgenomic SARS-CoV-2 RNAs are 7-methylguanosine capped

Coronavirus genomic RNA is presumed to be m^7^G capped, though there is little direct evidence to support this (6–8). Therefore, we began by confirming which SARS-CoV-2 viral RNAs are m^7^G capped.

**Figure 1.**
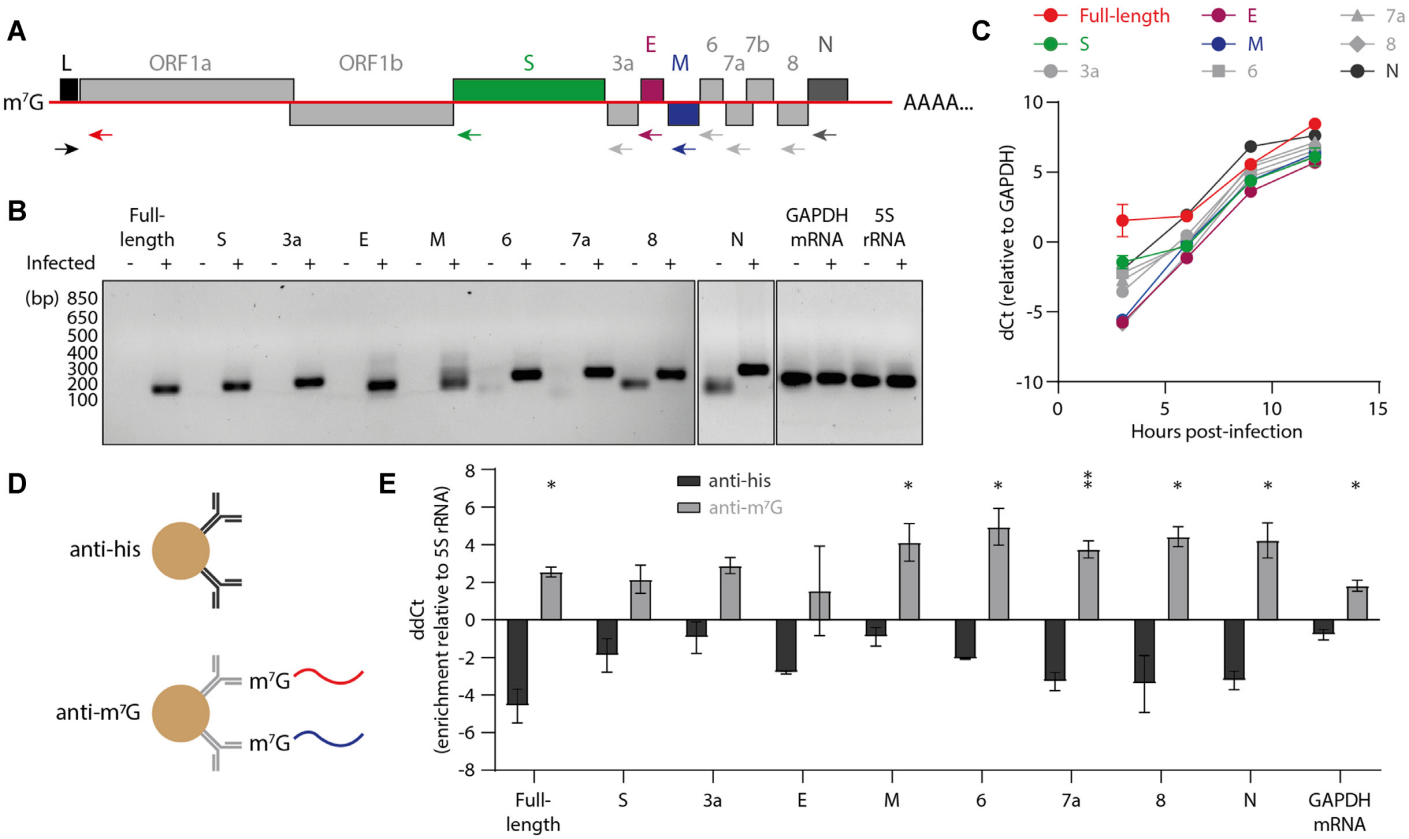
SARS-CoV-2 viral RNAs are 7-methylguanosine capped. (**A**) Schematic of the full-length SARS-CoV-2 genome with RT-qPCR primer binding sites indicated with arrows. (**B**) RT-qPCR was performed on total RNA from mock infected cells and cells 12 hours post-infection with SARS-CoV-2. Reaction products were resolved by agarose gel electrophoresis. Off-target amplicons from uninfected cells are visible for 8 and N sgRNA primers at high cycle numbers. (**C**) Vero cells were infected with SARS-CoV-2 and viral RNA accumulation was measured by RT-qPCR. Quantification of RNA levels is from *n* = 2 independent infections, data are mean ± s.e.m. (**D**) Schematic of RNA IP using an anti-m^7^G antibody, with an anti-his tag antibody as a negative control. (**E**) m^7^G capped RNA was immunoprecipitated from the total RNA of SARS-CoV-2 infected cells and quantified by RT-qPCR. RNA levels were normalised to uncapped 5S rRNA and the input was set to 0, such that a positive value indicates enrichment. Quantification of RNA enrichment is from n = 2 independent infections, data are mean ± s.e.m. Anti-his and anti-m^7^G values were compared by unpaired two-tailed *t*-test. *P < 0.05, **P < 0.01.

To achieve this we established a reverse-transcription (RT)-qPCR method to detect full-length genomic and subgenomic viral RNAs, and using this approach could detect all positive-sense viral RNA species in infected cells with the exception of 7b subgenomic RNA (Figure 1A, B) (28). As expected, all viral RNAs examined accumulated rapidly between 3 and 12 h post-infection with SARS-CoV-2 (Figure 1C). To identify m^7^G capped RNAs we performed immunoprecipitation (IP) on RNA extracted from SARS-CoV-2-infected cells using an anti-m^7^G antibody, with an anti-his tag antibody as a negative control (Figure 1D). Full-length viral RNA was significantly enriched compared to uncapped 5S rRNA by the anti-m^7^G IP, as were the M, 6, 7a, 8 and N subgenomic RNAs (Figure 1E). S, 3a and E subgenomic RNAs also appeared enriched by the IP, although were not statistically significant. These data provide direct evidence that genomic and subgenomic SARS-CoV-2 viral RNAs are m^7^G capped.

### Nsp12 has guanylyltransferase activity

Synthesising m^7^G capped RNA requires several distinct enzymatic activities, which are well characterised for coronaviruses apart from the viral GTase (4). We therefore aimed to identify and characterise the SARS-CoV-2 GTase.

**Figure 2.**
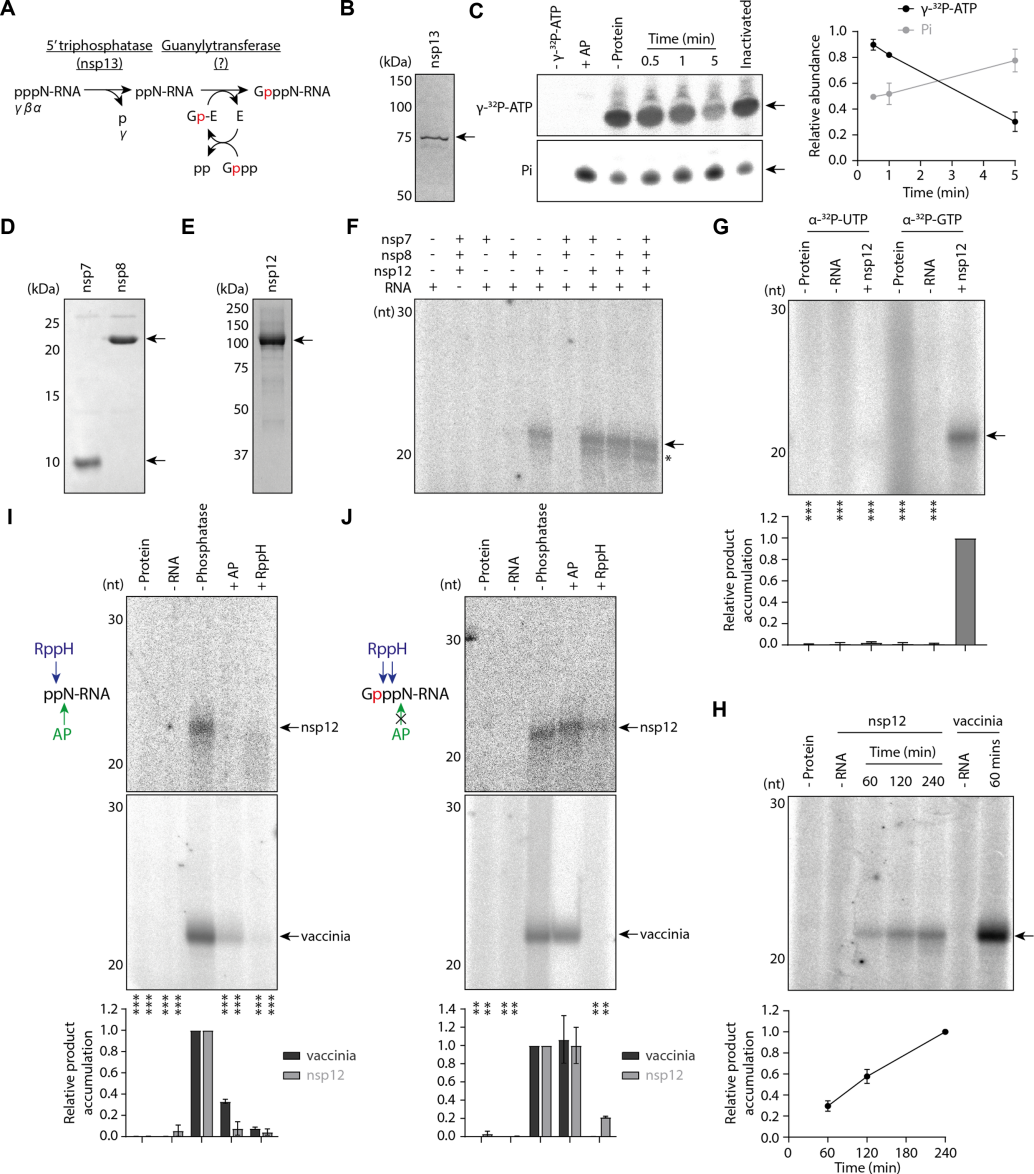
Nsp12 has guanylyltransferase activity. (**A**) Schematic of nsp13 5′ triphosphatase activity followed by a canonical GTase reaction mechanism, which consists of nucleotidylation of the enzyme (E) with GTP (Gppp), then transfer of GMP (Gp) to a diphosphorylated RNA substrate (ppN-RNA). The ^32^P isotope of α-^32^P-GTP is indicated in red. (**B**) Purified SARS-CoV-2 nsp13 was visualised by SDS PAGE, the arrow indicates the His6-ZBasic-tagged nsp13 band. (**C**) 5′ triphosphatase activity of purified nsp13 was tested by incubation with γ-^32^P-ATP for the indicated amount of time. The γ-^32^P-ATP substrate and inorganic phosphate (Pi) product were visualised by denaturing PAGE and autoradiography, arrows indicate the anticipated bands (left). AP was used as a positive control, and as a negative control nsp13 was inactivated by heating to 70°C for 5 min prior to the reaction, then was incubated with the γ-^32^P-ATP substrate for 5 mins. Quantification of γ-^32^P-ATP substrate and Pi product is from *n* = 2 independent reactions, data are mean ± s.e.m. (right). Pi present in the untreated γ-^32^P-ATP stock was subtracted from all samples during quantification. (**D**) SARS-CoV-2 nsp7 and nsp8 were expressed and purified from *E. coli* cells and visualised by SDS PAGE. Arrows indicate bands corresponding to the proteins of interest. (**E**) SARS-CoV-2 nsp12 was expressed and purified from Sf9 cells and visualised by SDS PAGE. The arrow indicates the purified nsp12 band. (**F**) The indicated SARS-CoV-2 proteins were incubated with α-^32^P-GTP and diphosphorylated RNA, then radiolabelled RNA products were visualised by denaturing PAGE and autoradiography. The arrow indicates the anticipated product, and the asterisk denotes a faster mobility product which could result from contaminating RNA kinase activity in some protein preparations, or decapping of the anticipated product by a phosphatase such as residual nsp13. (**G**) Nsp12 was incubated with diphosphorylated RNA and α-^32^P-UTP or α-^32^P-GTP, then radiolabelled RNA products were visualised by denaturing PAGE and autoradiography. The arrow indicates the anticipated product (top). Quantification of the product is from n = 3 independent reactions (bottom). Data are mean ± s.e.m., analysed by one-way ANOVA. ***P<0.01. (**H**) Nsp12 or vaccinia capping enzyme were incubated with α-^32^P-GTP and diphosphorylated RNA for the indicated amount of time. Radiolabelled RNA products were visualised by denaturing PAGE and autoradiography (left), the arrow indicates the anticipated product. Quantification of the product is from *n* = 3 independent reactions, data are mean ± s.e.m. (right). (**I**) Schematic of AP and RppH activity on the diphosphorylated RNA substrate (left). Diphosphorylated RNA was treated with AP or RppH, then incubated with nsp12 or vaccinia capping enzyme and α-^32^P-GTP (top). The arrow indicates the anticipated product. Quantification of the product is from *n* = 2 independent reactions (bottom). Data are mean ± s.e.m., analysed by one-way ANOVA. ***P < 0.001. (**J**) Schematic of AP and RppH activity on the capped RNA product (left). Diphosphorylated RNA was incubated with nsp12 or vaccinia capping enzyme and α-^32^P-GTP, then reaction products were treated with AP and RppH (top). The arrow indicates the anticipated product. Quantification of the product is from *n* = 2 independent reactions (bottom). Data are mean ± s.e.m., analysed by one-way ANOVA. **P < 0.01.

GTases are responsible for covalently linking GTP to diphosphorylated RNA to produce a cap-like structure (GpppN-RNA), and nsp13 is thought to generate the diphosphorylated RNA substrate by acting as a 5′ triphosphatase (Figure 2A) (9,29). We therefore confirmed the 5′ triphosphatase activity of purified nsp13, then used it to produce a diphosphorylated 20nt model RNA substrate for GTase reactions (Figure 2B, C).

As nsp12 has previously been implicated as a GTase, we expressed and purified SARS-CoV-2 nsp12 along with its RdRp accessory proteins nsp7 and nsp8 (Figure 2D, E) (13). To test for GTase activity we incubated the diphosphorylated 20nt RNA with nsp7, nsp8, nsp12 and α-^32^P-GTP, then separated the reaction products by denaturing PAGE (Figure 2F). In reactions containing nsp12 we observed a radiolabelled product running slightly slower than the 20nt marker, which was dependent on the presence of diphosphorylated RNA. A previous study indicated that the nsp12 GTase could utilise either a GTP or UTP substrate, therefore we also performed reactions using α-^32^P-UTP; in this case we did not observe any radiolabelled product, indicating that the reaction is nucleotide-specific (Figure 2G) (13).

To confirm that the radiolabelled product resulted from GTase activity we compared the activity of nsp12 with vaccinia capping enzyme, a known GTase (30). Under the same conditions vaccinia capping enzyme synthesized a radiolabelled product with the same mobility as that made by nsp12 (Figure 2H). We then performed enzymatic digestions of the substrate and product RNAs. First, we treated the substrate RNA with alkaline phosphatase (AP) or RNA 5′ pyrophosphohydrolase (RppH) to produce dephosphorylated and monophosphorylated RNAs respectively, then performed GTase reactions (Figure 2I). Vaccinia capping enzyme and nsp12 were both unable to efficiently use these RNAs as substrates, indicating that diphosphorylated RNA is required. Second, after running GTase reactions we treated reaction products with AP or RppH (Figure 2J). The products made by vaccinia capping enzyme and nsp12 were not sensitive to AP, but were degraded by RppH. This enzymatic profile is characteristic of a GTase product; the cap-like structure protects 5′ phosphates from AP, however, RppH can cleave internally in the 5′ triphosphate of the GpppN-RNA product (31). Collectively, these data confirm that SARS-CoV-2 nsp12 has GTase activity *in vitro*.

### The nsp12 NiRAN domain is responsible for guanylyltransferase activity

**Figure 3.**
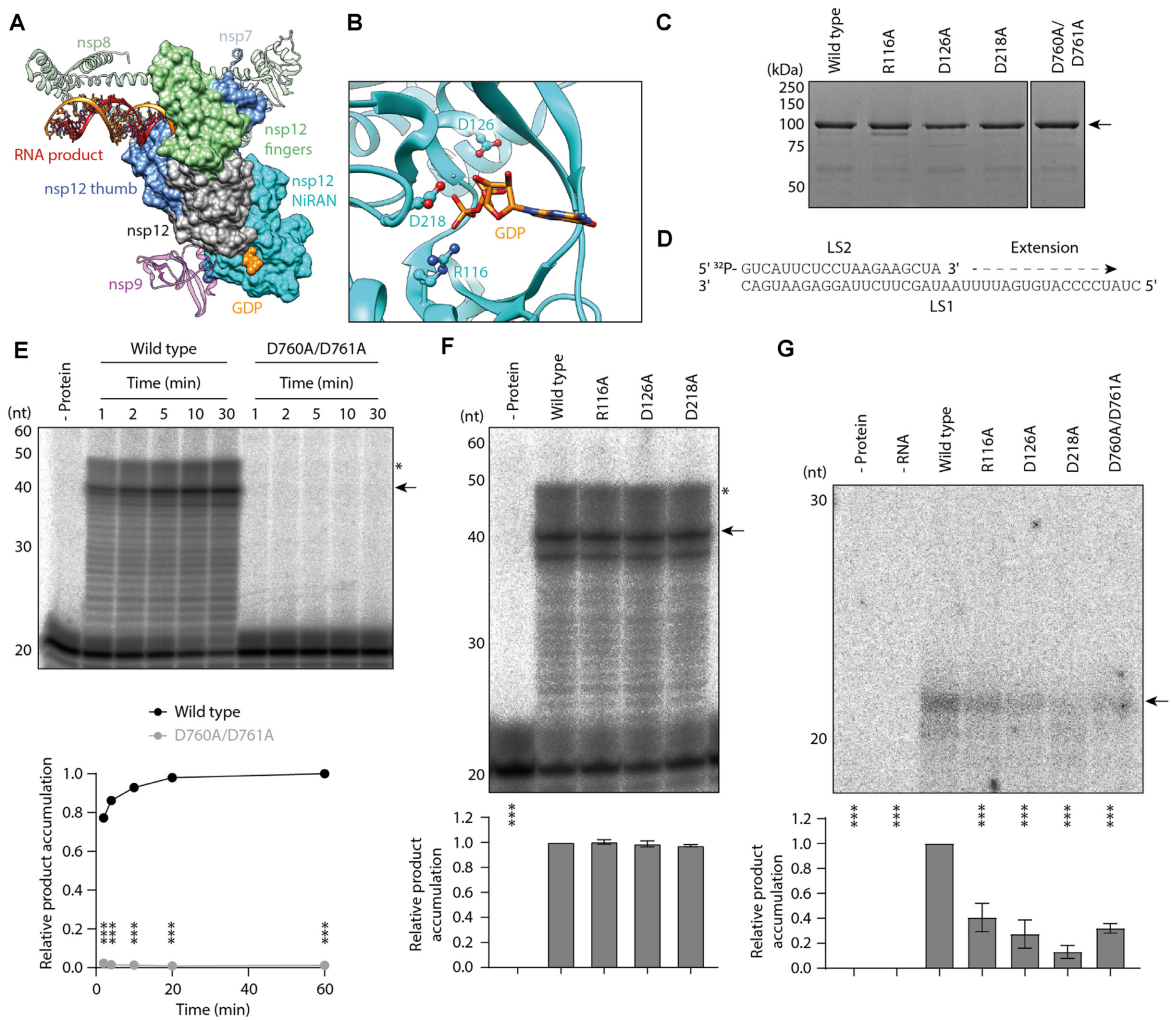
Mutations in the nsp12 NiRAN domain disrupt guanylyltransferase activity. (**A**) Structure of the SARS-CoV-2 nsp7/8/12 complex bound to RNA product, nsp9 and GDP (PDB: 7CYQ). Nsp12 (grey) is shown as a surface with the thumb and fingers subdomains highlighted in blue and green respectively. In this structure the NiRAN domain (cyan) at the nsp12 N-terminus is bound to GDP (orange), while nsp7 and nsp8 (turquoise and green ribbons) facilitate RNA product (orange/red) binding. (**B**) Close-up view of GDP bound to the nsp12 NiRAN domain with key amino acid residues highlighted, including D218 which coordinates an Mg^2+^ ion (silver). (**C**) Mutant nsp12 proteins purified from Sf9 cells were visualised by SDS PAGE. The arrow indicates the purified nsp12 band. (**D**) Schematic of the 40nt RNA template (LS1) and 20nt radiolabelled RNA primer (LS2). (**E**) Wild type or D760A/D761A mutant nsp12 was incubated with nsp7 and nsp8, then tested for RNA polymerase activity on the LS1/LS2 RNA template. Reaction products were resolved by denaturing PAGE and autoradiography (top). The arrow indicates the anticipated 40nt RNA product, and the asterisk denotes incompletely denatured RNA which has slower mobility on the gel. Quantification of the 40nt RNA product is from *n* = 3 independent reactions (bottom). Data are mean ± s.e.m., analysed by two-way ANOVA. ***P < 0.001. (**F**) Mutant nsp12 proteins were tested for RNA polymerase activity in the presence of nsp7, nsp8 and LS1/LS2 RNA template (top). The arrow indicates the anticipated 40nt RNA product, and the asterisk denotes incompletely denatured RNA which has slower mobility on the gel. Quantification of the 40nt product is from *n* = 3 independent reactions (bottom). Data are mean ± s.e.m., analysed by one-way ANOVA. ***P < 0.001. (**G**) Mutant nsp12 proteins were tested for GTase activity using α-^32^P-GTP and a diphosphorylated RNA substrate, the arrow indicates the anticipated product (top). Quantification of the product is from *n* = 3 independent reactions (bottom). Data are mean ± s.e.m., analysed by one-way ANOVA. ***P < 0.001.

Next, we aimed to identify the GTase active site in nsp12. A recent cryo-EM study showed that the nsp12 NiRAN domain can bind to GDP, so we hypothesised that this nucleotide binding pocket could be important for GTase activity (Figure 3A, B) (13). We therefore made alanine substitutions at nsp12 amino acid residues R116, D126 and D218, which are located in the nucleotide binding pocket and have previously been identified as important for SARS-CoV growth (Figure 3C) (17).

To first examine the effect of these mutations on the RNA polymerase activity of nsp12, we established an assay to measure the extension of a 20 nucleotide (nt) primer (LS2) along a 40nt RNA template (LS1) (Figure 3D) (21). In the presence of RNA template and rNTPs the wild type nsp7/8/12 complex was able to extend the LS2 primer to produce a 40nt major product; however, a complex of nsp7/8/12 with D760A/D761A mutations in the nsp12 RdRp active site was unable to extend the LS2 primer (Figure 3E). When we tested the effect of the nsp12 NiRAN domain mutations none disrupted RNA polymerase activity in the presence of nsp7 and nsp8 (Figure 3F).

In contrast, when we performed GTase reactions using the mutant nsp12 proteins we found that all NiRAN domain mutations significantly reduced GTase activity (Figure 3G). The D218A mutation was the most inhibitory, reducing GTase activity to ∼10% of wild type nsp12. This result is consistent with structural data which show that D218 coordinates a magnesium ion in the NiRAN domain, and therefore has a key role in nucleotide binding (Figure 3B) (13,23). Interestingly, the D760A/D761A RdRp active site mutation also reduced GTase activity to ∼40% of wild type nsp12.

**Figure 4.**
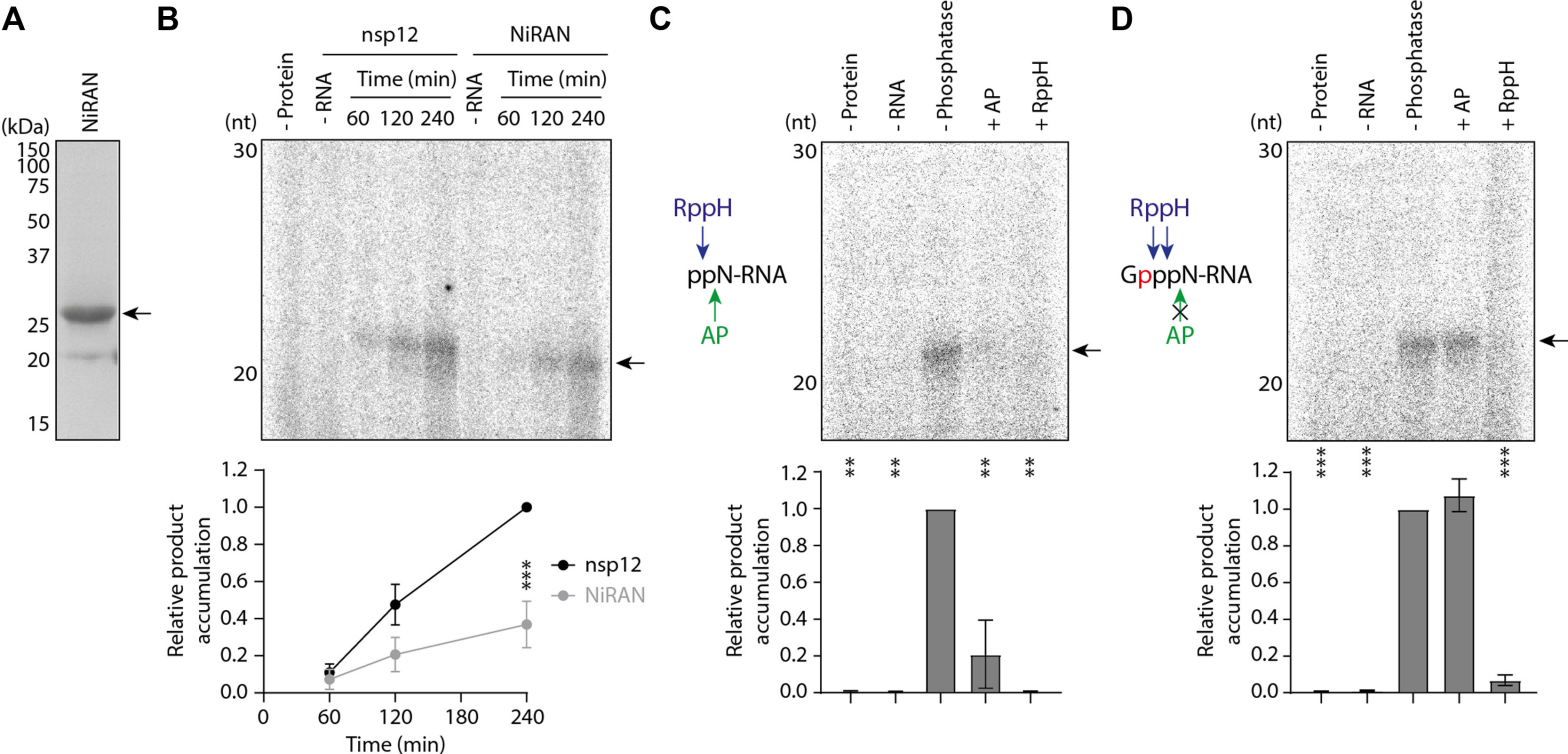
The nsp12 NiRAN domain alone is a guanylyltransferase enzyme. (**A**) The NiRAN domain from SARS-CoV-2 nsp12 was expressed and purified from *E. coli*, then visualised by SDS PAGE. The arrow indicates the purified NiRAN domain band. (**B**) Full-length nsp12 and the purified NiRAN domain were tested for GTase activity using α-^32^P-GTP and a diphosphorylated RNA substrate, the arrow indicates the anticipated product (top). Quantification of the product is from *n* = 3 independent reactions (bottom). Data are mean ± s.e.m., analysed by two-way ANOVA. ***P < 0.001. (**C**) Schematic of AP and RppH activity on the diphosphorylated RNA substrate (left). Diphosphorylated RNA was treated with AP or RppH, then incubated with purified NiRAN domain and α-^32^P-GTP (right). The arrow indicates the anticipated product. Quantification of the product is from n = 2 independent reactions (bottom). Data are mean ± s.e.m., analysed by one-way ANOVA. **P < 0.01. (**D**) Schematic of AP and RppH activity on the capped RNA product (left). Diphosphorylated RNA was incubated with purified NiRAN domain and α-^32^P-GTP, then reaction products were treated with AP and RppH (right). The arrow indicates the anticipated product. Quantification of the product is from *n* = 2 independent reactions (bottom). Data are mean ± s.e.m., analysed by one-way ANOVA. ***P < 0.001.

To then investigate whether the isolated NiRAN domain has GTase activity, we expressed and purified the SARS-CoV-2 NiRAN domain and performed GTase reactions alongside full-length nsp12 (Figure 4A, B). The NiRAN domain alone was able to produce a radiolabelled product with the same mobility as that of full-length nsp12, but with ∼30% of the activity. To confirm that the isolated NiRAN domain performs a GTase reaction, we carried out enzymatic digestions of substrate and product RNAs. The NiRAN domain did not synthesise any radiolabelled product using dephosphorylated or monophosphorylated substrate RNA, produced by treatment of the diphosphorylated RNA with AP or RppH respectively (Figure 4C). Furthermore, the radiolabelled product made by the NiRAN domain reaction was sensitive to degradation by RppH but not AP (Figure 4D). These results are comparable to those of full-length nsp12 and vaccinia capping enzyme, confirming that the isolated NiRAN domain is an active GTase enzyme.

### *In vitro* reconstitution of 7-methylguanosine cap synthesis

**Figure 5.**
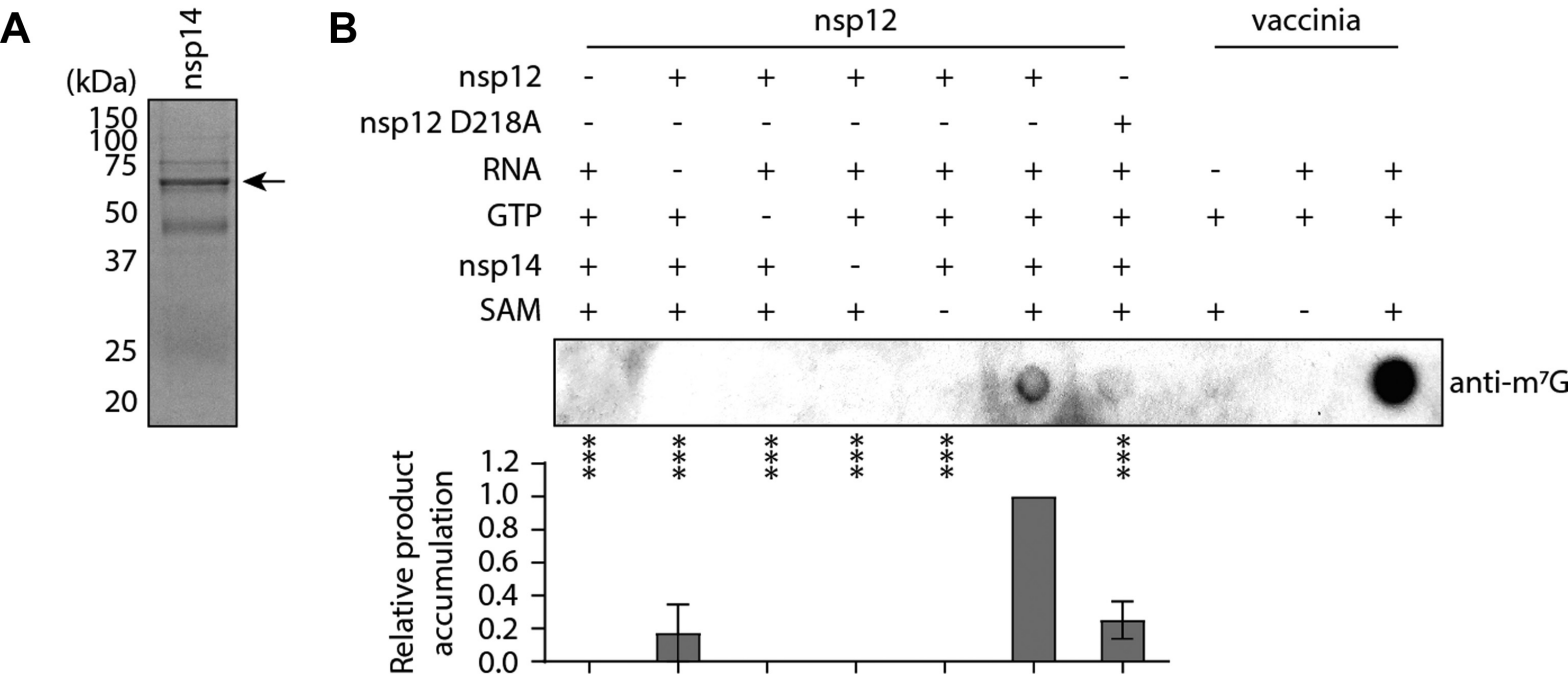
*In vitro* reconstitution of 7-methylguanosine cap synthesis. (**A**) SARS-CoV-2 nsp14 was expressed and purified from *E. coli*, then visualised by SDS PAGE. The arrow indicates the purified nsp14 band. (**B**) Nsp12 GTase reactions were treated with nsp14 and SAM, then reaction products were visualised by dot-blotting using an anti-m^7^G antibody (top). Quantification of the product is from n = 3 independent reactions (bottom). Data are mean ± s.e.m., analysed by one-way ANOVA. ***P < 0.001.

To produce an m^7^G capped viral RNA the product of the nsp12 GTase reaction must be subsequently methylated by the nsp14 N7-methyltransferase (10). To confirm that the nsp12 GTase product is a substrate for nsp14, we incubated GTase reactions with purified SARS-CoV-2 nsp14 and the methyl donor S-adenosylmethionine (SAM) (Figure 5A). We then visualized reaction products by blotting with the same anti-m^7^G antibody used for viral RNA IP from SARS-CoV-2 infected cells (Figure 5B). Only in the presence of all reaction components did nsp12 and nsp14 produce m^7^G capped RNA, and synthesis of this product was significantly reduced by the nsp12 D218A mutation. These results demonstrate that nsp14 can N7-methylate the nsp12 GTase product, which is consistent with the nsp12 GTase being part of the coronavirus RNA capping pathway.

### Remdesivir triphosphate can inhibit guanylyltransferase activity

**Figure 6.**
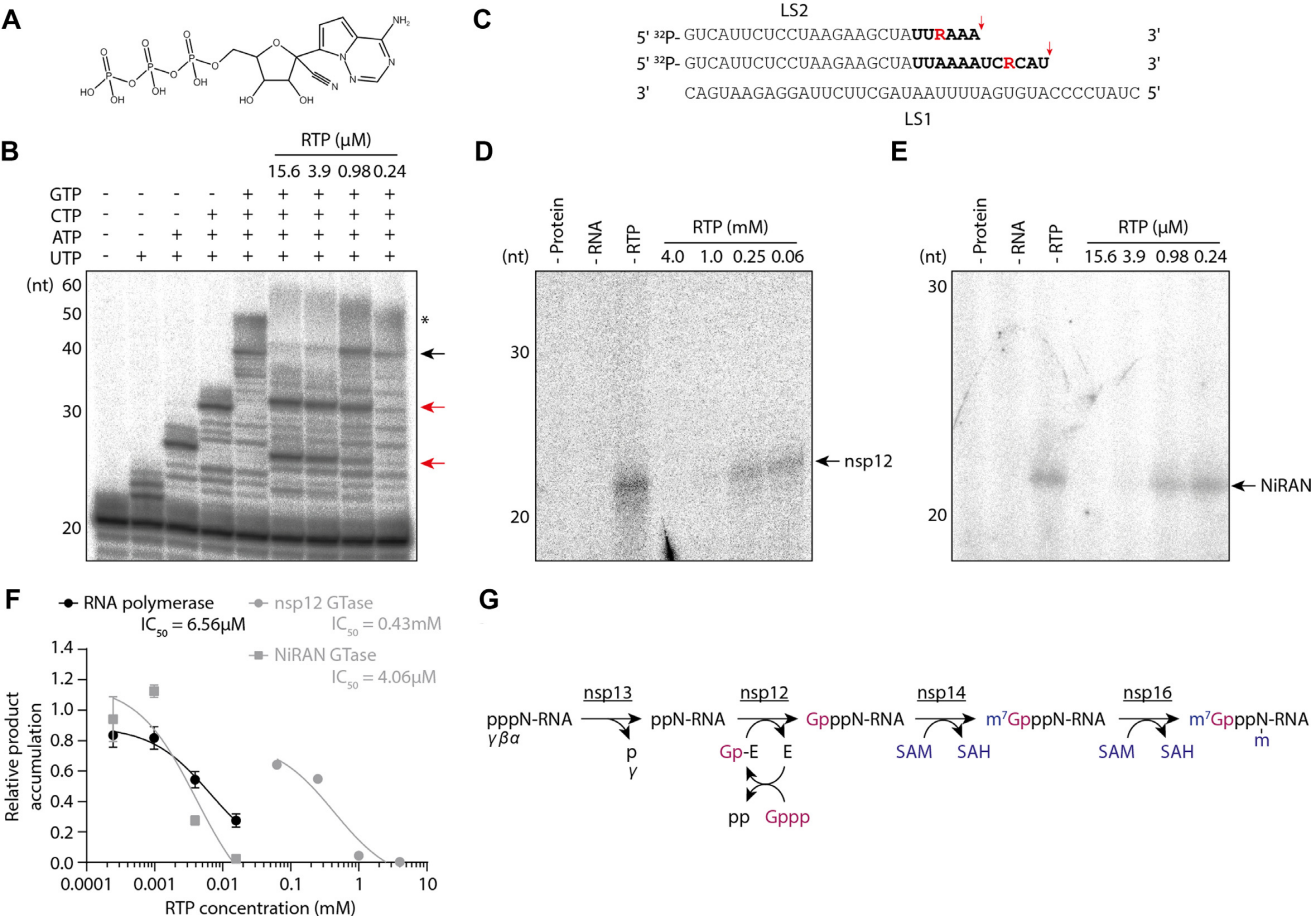
Inhibition of SARS-CoV-2 RNA polymerase and guanylyltransferase functions by remdesivir triphosphate and a model for coronavirus RNA capping. (**A**) Structure of remdesivir triphosphate, active metabolite of remdesivir. (**B**) Remdesivir triphosphate was titrated into RNA polymerase reactions containing nsp7/8/12 and LS1/LS2 RNA template. The black arrow indicates the anticipated 40nt RNA product, red arrows indicate major premature termination products, and the asterisk denotes incompletely denatured RNA which has slower mobility on the gel. (**C**) Schematic of the LS1/LS2 RNA template suggesting possible identities of major premature termination products. Incorporated bases are shown in bold, with remdesivir triphosphate residues in red. Red arrows indicate the point of termination. (**D**) Remdesivir triphosphate was titrated into nsp12 GTase reactions with α-^32^P-GTP and a diphosphorylated RNA substrate, the arrow indicates the anticipated product. (**E**) Remdesivir triphosphate was titrated into NiRAN domain GTase reactions, the arrow indicates the anticipated product. (**F**) Inhibition curves for remdesivir triphosphate in SARS-CoV-2 RNA polymerase and GTase reactions. Quantifications of the 40nt RNA product, nsp12 GTase product and NiRAN GTase product are each from *n* = 3 independent reactions. Data are mean ± s.e.m., fit to dose-response inhibition curves by nonlinear regression in GraphPad Prism 9. (**G**) Model for coronavirus RNA capping. The γ-phosphate of triphosphorylated viral RNA is removed by the 5′ triphosphatase activity of nsp13. The nsp12 NiRAN domain GTase links diphosphorylated viral RNA to GTP (purple), which involves a GMP-enzyme intermediate. Nsp14 methylates GTP at the N7 position using SAM (blue) as a methyl donor, and producing SAH. Nsp16 methylates the 2’ hydroxyl group of the nucleotide at position 1 of the RNA, generating viral RNA with a cap-1 structure as the final product.

Our data show that the NiRAN domain of SARS-CoV-2 nsp12 is a GTase, a reaction which requires nucleotide binding and therefore could be inhibited by nucleotide analogue drugs. One such nucleotide analogue is remdesivir triphosphate, active metabolite of the drug remdesivir which potently inhibits SARS-CoV-2 growth in cell culture (Figure 6A) (32).

We first tested the inhibitory effect of remdesivir triphosphate on RNA polymerase activity using the nsp7/8/12 complex where, in the presence of 1 μM rNTPs, the drug inhibited activity with an IC_50_ of 6.56 μM (Figure 6B, F). Truncated RNA products observed at inhibitory concentrations could be explained by the drug causing delayed chain termination 3nt downstream of certain uracil residues in the LS1 template, which is consistent with several previous studies (Figure 6C) (33–36).

We then titrated remdesivir triphosphate into nsp12 GTase reactions, where the drug also inhibited product accumulation with an IC_50_ of 0.43 mM (Figure 6D, F). To confirm that remdesivir triphosphate inhibits the GTase reaction by binding to the NiRAN domain, we then titrated the drug into reactions performed using the NiRAN domain alone (Figure 6E, F). Under these conditions the drug also inhibited product accumulation but with a much lower IC_50_ of 4.06 μM. Together, these data demonstrate that remdesivir triphosphate can inhibit the GTase function of SARS-CoV-2 nsp12 in addition to RNA polymerase activity *in vitro*.

## DISCUSSION

Coronaviruses are important human pathogens, but their mechanisms of viral RNA synthesis remain poorly understood. Here we provide insight into coronavirus RNA capping by showing that the NiRAN domain of SARS-CoV-2 nsp12 is a GTase enzyme involved in m^7^G cap synthesis.

This finding allows us to propose a model for the enzymatic processes in coronavirus RNA capping (Figure 6G). Nascent genomic or subgenomic viral RNA is the substrate for cap synthesis and, while the mechanism of viral RNA synthesis initiation remains unclear, reports of *de novo* initiation suggest that nascent viral RNA is 5′ triphosphorylated (21,37–39). The γ-phosphate of nascent viral RNA is removed by the 5′ triphosphatase activity of nsp13, which interacts directly with the nsp7/8/12 complex, and the resulting diphosphorylated viral RNA can then act as a substrate for the nsp12 GTase (9,23). Our data confirm that the GTase product can then be methylated by nsp14, which transfers a methyl group from SAM to N7 of guanine to produce m^7^G capped viral RNA and S-adenosylhomocysteine (SAH) as a by-product (Figure 5B) (10,40). Nsp16 carries out a second methylation on the 2’ hydroxyl of the nucleotide at position +1, producing viral RNA with a cap-1 structure (12,41,42). Higher eukaryotes have mRNA with cap-1, so this ensures that viral RNA is not recognised as non-self (43).

Canonical GTases use a nucleotidylated enzyme intermediate (Gp-E) to transfer GMP to a diphosphorylated RNA substrate (29). This intermediate has been previously characterised for the nsp12 proteins of SARS-CoV-2 and human coronavirus 229E (HCoV-229E), as well as the RNA polymerase of the related equine arteritis virus (EAV), meaning SARS-CoV-2 nsp12 likely utilises a similar mechanism (13,17,18). The latter study concluded that nucleotidylation occurs in the NiRAN domain, which is consistent with our finding that the SARS-CoV-2 nsp12 NiRAN domain contains the GTase active site (Figure 4B) (17). However, we also find that full-length nsp12 is more efficient in the GTase reaction than the isolated NiRAN domain and mutations in the RdRp domain reduce GTase activity (Figure 3G). These results suggest that the nsp12 RdRp domain indirectly supports the GTase reaction, possibly by binding to the RNA substrate.

Nucleotidylation of the NiRAN domain with UTP is more efficient than with GTP, although here we show that α-^32^P-UTP cannot be transferred to a diphosphorylated RNA substrate (Figure 2G) (17,18). This finding is consistent with other GTase enzymes, including vaccinia capping enzyme, but contradicts a previous study which reported SARS-CoV-2 nsp12 uridylyltransferase (UTase) activity (13,29,44). As the previous study did not directly compare UTP and GTP substrates, it is possible that nsp12 exhibits an extremely weak UTase activity that we were unable to detect upon comparison with the GTase. Further work will be required to determine the absolute enzymatic activity of the GTase and proposed UTase reactions.

If the only function of the NiRAN domain is as a GTase enzyme then why can it also nucleotidylate with UTP? Possibly because in addition to self-nucleotidylation, which we propose is a GTase reaction intermediate, the SARS-CoV-2 nsp12 NiRAN domain has been implicated in the *trans*-nucleotidylation of nsp8 and nsp9 with UTP (18–20). Nsp8 nucleotidylation with UTP has been shown to allow protein-primed RNA synthesis *in vitro* through a mechanism reminiscent of picornavirus RNA synthesis using nucleotidylated VPg (Viral Protein genome-linked) (19). The function of nsp9 nucleotidylation is less clear, with current studies proposing its involvement in either protein-primed RNA synthesis or the regulation of m^7^G cap synthesis. Nsp9 nucleotidylation is also not dependent on the nsp12 RdRp domain, possibly because RNA binding is not required for this reaction (18,20). Interestingly, all of these proposed activities utilise the same nsp12 D218 amino acid residue as the GTase reaction, raising the possibility that the NiRAN domain uses a single active site to carry out several diverse functions in coronavirus RNA synthesis (Figure 3G) (19,20). However, before this can be concluded, each of the functions proposed for the NiRAN domain including GTase activity require further *in vivo* evidence.

Viral RNA synthesis is central to the SARS-CoV-2 viral life cycle, and is the target of nucleoside analogue drugs such as remdesivir. Our *in vitro* assays indicate that remdesivir triphosphate inhibits SARS-CoV-2 RNA polymerase activity with an IC_50_ of 6.56 μM, higher than the 0.77 μM IC_50_ reported for SARS-CoV-2 infections in cell culture (Figure 6F) (32). This could be due to the *in vitro* assay not fully reflecting the RNA polymerase activity requirements *in vivo*; for example, in an infection the RNA polymerase must processively synthesise products of up to 30 kb without incorporating the drug (3). Alternatively, the lower IC_50_ of remdesivir *in vivo* could be explained by a dual mechanism of action, as we find that remdesivir triphosphate can also inhibit nsp12 GTase activity *in vitro* (Figure 6F). This is consistent with a previous report that remdesivir triphosphate can also competitively inhibit nsp9 nucleotidylation by the SARS-CoV-2 nsp12 NiRAN domain (20). Interestingly, we observe that remdesivir triphosphate has a much lower IC_50_ in the isolated NiRAN domain GTase reaction than in the full-length nsp12 reaction (Figure 6F). While we do not understand the reason for this difference in IC_50_, these data raise the possibility that the NiRAN domain could be targeted by other nucleotide analogues. This suggestion is substantiated by another study which demonstrated that nsp8 *trans*-nucleotidylation by the SARS-CoV-2 nsp12 NiRAN domain can be inhibited by nucleotide analogue AT-9010, which binds in the NiRAN domain active site (19). The GTase activity assays we have established here could be useful as tools to assess the potency of such compounds.

In summary, we have demonstrated that SARS-CoV-2 nsp12 is a GTase enzyme and identified the active site as the nsp12 NiRAN domain. These findings not only improve understanding of coronavirus RNA synthesis, but also highlight a new target for novel or repurposed antiviral drugs against SARS-CoV-2.

## DATA AVAILABILITY

Source data as well as plasmids are available upon request.

## Notes

Present address: Alexander P. Walker, School of Cellular and Molecular Medicine, Faculty of Life Sciences, University of Bristol, Bristol BS8 1TD, UK.
